# Database of Periodic DNA Regions in Major Genomes

**DOI:** 10.1155/2017/7949287

**Published:** 2017-01-15

**Authors:** Felix E. Frenkel, Maria A. Korotkova, Eugene V. Korotkov

**Affiliations:** ^1^Institute of Bioengineering, Research Center of Biotechnology of the Russian Academy of Sciences, Leninsky Ave. 33, bld. 2, 119071 Moscow, Russia; ^2^National Research Nuclear University “MEPhI”, Kashirskoe Shosse 31, Moscow 115409, Russia

## Abstract

*Summary*. We analyzed several prokaryotic and eukaryotic genomes looking for the periodicity sequences availability and employing a new mathematical method. The method envisaged using the random position weight matrices and dynamic programming. Insertions and deletions were allowed inside periodicities, thus adding a novelty to the results we obtained. A periodicity length, one of the key periodicity features, varied from 2 to 50 nt. Totally over 60,000 periodicity sequences were found in 15 genomes including some chromosomes of the* H. sapiens* (partial),* C. elegans*,* D. melanogaster,* and* A. thaliana* genomes.

## 1. Introduction

Periodicity is one of the sequences' structural regularities and is widely represented in the amino and DNA sequences [1]. A periodicity is considered as latent, if the similarity between any two periods is not statistically significant or if it belongs to the twilight zone [2]. Perfect periodicity can transform into latent periodicity, if it accumulates over 1.0 mutation per nucleotide in the studied DNA sequence [3] and a certain number of insertions and deletions of bases (this number > 1 per each period). Availability of a large number of base substitutions, insertions, and deletions in each period is regarded to represent the latent periodicity property. The distinctive property of latent periodicity is that it cannot be detected by pairwise comparison of the nucleotide sequences. However, latent periodicity can be found, if a mathematical method is applied to directly detect the alignment of nucleotide sequences without constructing pairwise alignments. The periods of a sequence with latent periodicity are sequences for multiple alignment that is statistically significant. The aim of this study was to apply the previously developed mathematical method [4, 5] and to detect the DNA sequences periodicity, as well as the latent periodicity, and to create the new data bank.

At present, there is a significant gap in the mathematical approaches developed in the periodicities search for symbolic and numeric sequences (sequence-based methods). Spectral approaches enable detection of the adequate “fuzzy” periodicity in nucleotide sequences without the nucleotides insertion(s) or deletion(s). In this case, the number of base substitutions can be over 1.0 per nucleotide [3]. Fourier transform, wavelet transform, information decomposition, and some other methods could be included within the spectral methods list [1, 3, 6–15]. However, these approaches have a significant limitation; they do not ensure detection of a periodicity with insertions and deletions. However, the DNA sequences obtain not only base substitutions, but also insertions and deletions in the course of evolution. Therefore, spectral methods are rarely used to study the DNA sequences of complete genomes. For this purpose, we need a mathematical method that would allow us to detect the sequences similarity that accumulates more than 1.0 base substitutions per nucleotide in the presence of the bases insertions and deletions, that is, to find the latent periodicity.

On the other hand, methods based on pairwise alignment or algorithmic methods are able to accurately detect insertions and deletions [16–20]. However, these methods cannot detect a latent periodicity in a situation, where the statistical significance of similarity between any two periodic sequences is insignificant [1, 21]. This happens due to the fact that the DNA sequences periodicity (with the number of periods greater than or equal to 4) is detected by pairwise similarity between the periods. In the absence of statistically significant pairwise similarity, these approaches are unable to find the latent periodicity. First of all, it involves algorithms and programs, such as TRF [16], Mreps [22], TRStalker [23], ATRHunter [24], T-REKS [25], IMEX [26, 27], CRISPRs [28], SWAN [29], and some others [30, 31], because similarity between the different periods is very low in case of latent periodicity. This leads to the lack of seeds and identical short strings.

The frequency matrix [32] or corresponding position weight matrix* M* could describe a sequence periodicity* S* with the* N* length [33]. Rows of this matrix are DNA bases, and the columns are the period positions. The DNA base* i* in position* j* of the period has weight *m*(*i*, *j*) and positions of the period vary from 1 to* n*. We create artificial periodic sequence* S*_1_ of the* N* length, which is 1,2,…, *n*, 1,2,…, *n*,…. The numbers are considered as symbols; and they correspond to the columns of matrix* M*. A certain frequency matrix and weight matrix *M*(20, *n*) correspond to the period equal to* n* in the sequence* S*. We can formulate the following problem. We have a sequence* S* with the length* N*. We should find the optimal weight matrix* M*_0_, where the local alignment of sequences* S*_1_ and* S* obtain the highest statistical significance. Under the statistical significance, the probability* P* determines that *F*_*r*_ > *F*_max_, where *F*_max_ is the maximum weight of a local alignment of sequences* S* and* S*_1_, using the optimal matrix* M*_0_. Here, *F*_*r*_ is the maximum weight of a local alignment of the randomly mixed sequence* S* and sequence* S*_1_ using the optimal matrix *M*_*r*_. It is always possible to set the threshold level of the probability* P*_0_ and, if the probability *P*(*F*_*r*_ > *F*_max_) is less than* P*_0_, then the revealed local alignment of sequences* S* and* S*_1_ using the optimum matrix* M*_0_ can be considered as statistically significant.

A local alignment algorithm could be used for the alignment of the DNA sequence* S* and the artificial periodic sequence* S*_1_ using the known weight matrix [34]. But we should find the optimal weight matrix* M*_0_. Previously we developed a mathematical approach for detecting the matrix* M*_0_, as well as a method for assessing the probability* P* [4, 5]. The periods multiple alignments were calculated by optimizing the PWM without using the pairwise alignments or the similarity search between periods. This approach allows us to detect latent periodicity that is the periodicity, which accumulated more than 1.0 base substitution and a number of the bases deletions or insertions within each study period. Periodicities with a smaller number of base substitutions are detected by this method without any problems. The developed algorithm was applied to search for periodicity with insertions and deletions in the different genomes. This study showed the presence of latent periodicity (over 1.0 mutation per nucleotide with insertions and deletions) in some chromosomes of the* H. sapiens* and* C. elegans*,* D. melanogaster,* and* A. thaliana* genomes, where the presence of periodicity was not previously discovered. The results are included in database, which is available online at the following website: http://victoria.biengi.ac.ru/indelper/.

## 2. Methods and Algorithms

### 2.1. Using Genetic Algorithm

Optimal weight matrix* M*_0_ for period* n* and for sequence* S* was calculated using the genetic algorithm. Genetic algorithm is a heuristic search algorithm for solving the optimization problems and is a form of the direct random search [35]. It is often used to optimize the functions of several variables. Usually, the problem is formalized, so that a solution could be found as a vector, where each element can be a bit, a number, or some other object. This vector is considered as an “organism.” Usually, a set of initial organisms are created randomly [36]. Each of these organisms was measured using the objective function, which is regarded as a “fitness function.” As a result, every organism was associated with a certain fitness value, which determines how well the organism solves the problem. Separate organisms were selected from this set of organisms (it can be called “generation”) for application of the “genetic operators” (“crossing” and “mutation,” taking into account the “fitness” value). The new organisms were received as a result of these operators application. The fitness value was also calculated for new organisms and then selection of the best organisms for the next generation was performed. This set of actions was repeated iteratively and thus simulated the “evolutionary process.” This process was allowed to continue for several life cycles (generations), before executing the termination criterion of the algorithm. Such a criterion can find either the global or suboptimal solutions or exhaustion of the number of generations released for evolution.

The organisms in our case are the periodicity weight matrices. This set is called *Q*_*n*_ or population. Each matrix has 4 rows and* n* columns. Matrix elements *m*(*i*, *j*) are certain numbers that show weight base* i* in column number* j*. A larger weight of the element *m*(*i*, *j*) corresponds to the high probability of the presence of the base* i* at position* j* of the period. As the assessment of fitness (objective function) for the organism (weight matrix* M*), the maximum value of the similarity function *F*_max_ was considered for the local alignment [38]. A local alignment was created between sequences* S*_1_ and* S,* using the weight matrix* M* to calculate the objective function. The calculation of *F*_max_ was performed for each organism (weight matrix* M*). The process was repeated after applying the genetic operators to the organisms. The process was terminated after a stable population was achieved; that is, the increase in the values of *F*_max_ was terminated. As a result, the matrix* M*_0_ was defined for the period length* n*, which obtains the greatest value *F*_max_ (*mF*_max_). The alignment of sequences* S*_1_ and* S* was well built using the matrix* M*_0_. The algorithm was repeated for* n* from 2 to 100. The method we used to reveal DNA periodicity sequences was previously developed and applied to amino acid sequences and the details could be found in the following studies [4, 5, 39]. It can effectively detect periodicity in the presence of insertions, deletions, and large number of substitutions of nucleotides (on the average, more than 1 per nucleotide). Until now methods, which could find periodicity under these conditions, are missing. Both terms and functions referred to here follow notations defined in the paper cited above.

### 2.2. Genome Analysis and Statistical Importance Detection Level

Periodicity sequences were detected in a 600 nt long sliding window (sequence* S*) that was shifted by 200 bp iteratively. In order to avoid interference with the triplet periodicity in the genome protein-coding regions, we skipped those windows that had statistically significant triplet periodicity* Z* value (see [40] for details) over 3.0. In the given window we found the position weight matrix* M* [1], which obtained the best local alignment that maximizes similarity score *F*_max_ [5, 39] for each tested period length* n* (from 2 to 50). In order to account for interwindow duplicates we filtered out intersecting sequences by choosing the one having larger *F*_max_ value (*mF*_max_).

**Figure 1:**
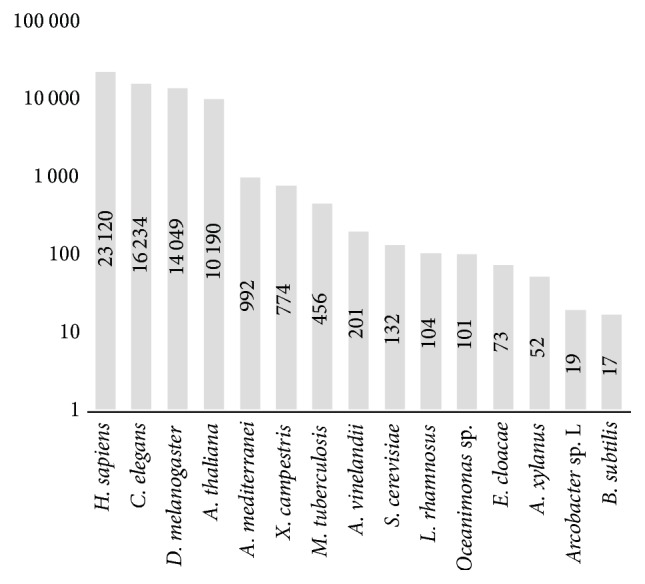
Distribution of found sequences with periodicity by organisms.

We evaluated the search statistical significance by finding the cutoff level for *mF*_max_. Numerical simulation via genome sequences shuffling showed that *mF*_max_ = 390.0 corresponds to the false positives ratio ≤ 5%. Altogether, we analyzed 15 major prokaryotic and eukaryotic genomes. Human genome was analyzed partially (from 19th to 22nd chromosomes) due to intensive algorithm calculation and time limitations. We are continuing the human genome analysis and the results in regard to the new chromosomes will be posted, as calculation is completed. We revealed 66,596 periodic sequences in the analyzed genomes.  Figure 1 shows distribution of the detected sequences throughout the organisms. The largest number of sequences was found in the human genome, but we could not completely analyze the genome. The complete human genome will be analyzed early in 2017.

## 3. Results and Discussion

Our results are published as a web database at http://victoria.biengi.ac.ru/indelper. It provides basic navigation within organisms and filtering using the periodicity length and periodicity significance score (*mF*_max_). Each periodicity sequence could be analyzed in detail by selecting it in query results. The page will provide data on genomic position, statistical significance of triplet periodicity (*Z*) [40], alignment against periodicity, periodicity consensus sequence, and periodicity weight matrix *M* [33].

**Figure 2:**
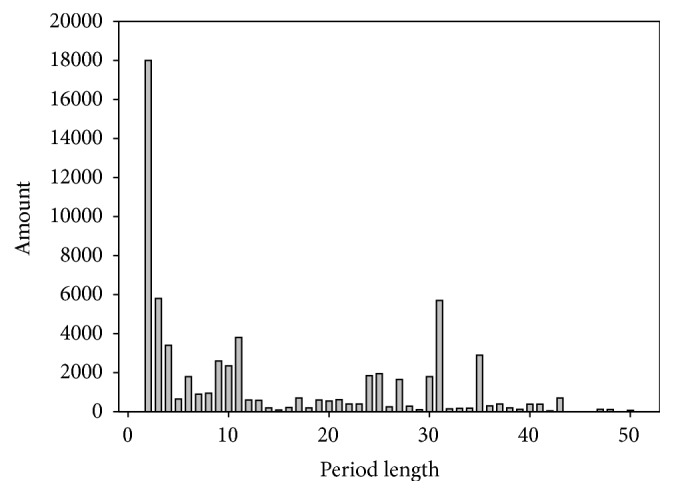
Distribution of found sequences by period length in all species.

Search composition by periodicity length is shown further in Figure 2. The heterogeneous nature of distribution is clearly distinguished and we can see a few peaks in the distribution. Firstly, a large number of sequences are observed within the periodicity length of 2 to 4 DNA bases. As we note below, it could be highly divergent microsatellite sequences. Secondly, a distinguished peak could be observed from 9 to 11 bases. Thirdly, some peaks are present in bases 31 and 35.

**Figure 3:**
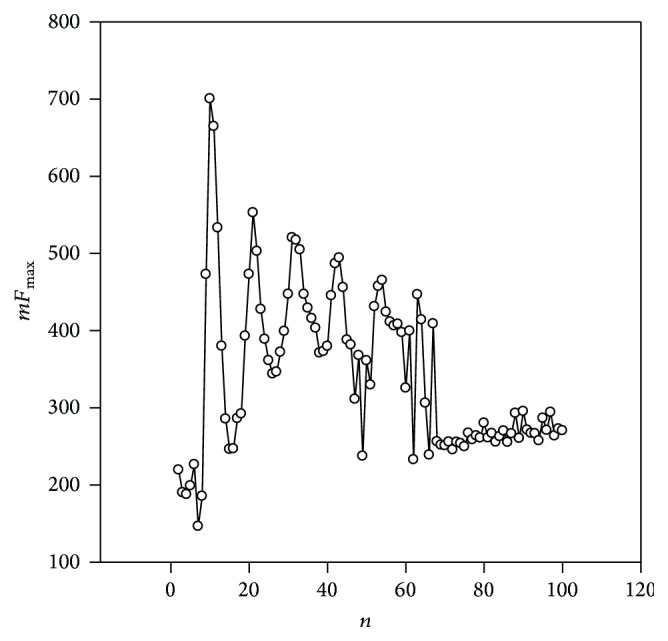
*mF*_max_(*n*) spectrum for fragment of the sequence NC_003074.1 from chromosome 3 of the* C. elegans* genome. The coordinates of fragment are 11055357–11055823.

In this study, two sequences obtaining periodicity were considered as the examples. The first sequence had a periodicity length of 10 nucleotides and this periodicity could be detected only in the presence of deletions or insertions. The spectrum of *mF*_max_(*n*) is shown in Figure 3. This region was found in the third chromosome of the* Arabidopsis thaliana* genome, in sequence NC_003279.8. *mF*_max_(4) = 726.31. This periodicity was not detected using the TRF [16], ATR hunter [24], Repfind [41], and Mreps [22] programs. T-REKs [25] found 11 periodicities equal to 10 nucleotides only. However, the 10 bp periodicity in the region with length equal to 466 bases was found by our method (more 46 periods). It is more than four times greater than the length found by the T-REKs program, and very high levels of statistical significance were found. TRStalker program [23] found 4 repeats with the length of 22 bases but did not find 11 base repeats. BWT program [42] found 3 repeats in the sequence with length of 10 bp. According to this study's estimates, *mF*_max_(4) = 726.31, and it corresponds to *P*(*mF*_max_ > 726.31) < 10^−50^, because the average value of *mF*_max_ for random sequences* Sr* is about 142.6 and *σ* ~ 52.3. The resulting alignment and the resulting matrix* M*_0_ could be received at http://victoria.biengi.ac.ru/cgi-bin/indelper/index.cgi. A consensus periodicity with length equal to 11 nucleotides is (A/C)AAG(A/G)(C/G)(T/G)TTTC. This periodicity was repeated more than 40 times in the region found and the periodicity equal to 11 bases obtained the highest statistical significance.

**Figure 4:**
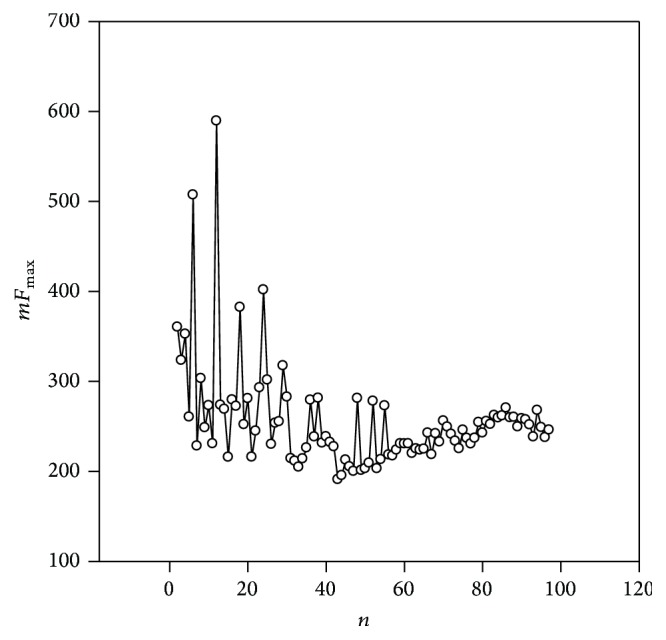
*mF*_max_(*n*) spectrum for fragment of the sequence AE014134.6 from chromosome 2*l* of the* Drosophila melanogaster* genome. The coordinates of fragment are 10643993–10644422.

The second example includes the sequence from chromosome 2*l* of the* Drosophila melanogaster* genome and obtains periodicity equal to 12 nucleotides. The sequence code is AE014134.6 and the periodicity sequence ranged from 10643993 to 10644422 bases.  Figure 4 shows the spectrum *mF*_max_(*n*). From this graph it is clear that the highest statistical significance obtained periodicity equal to 12 nucleotides and *mF*_max_(11) = 592.0. The discovered sequence obtained the length equal to 430 nucleotides and contained more than 35 highly divergent 12 base repeats. TRF [16], Mreps [22], TRStalker [23], Repfind [41], ATR hunter [24], and BWTRs [42] programs did not find repeats equal to 11 nucleotides in the sequence. T-REKs program [25] revealed 7 repeats having length equal to 5 bases and did not find repeats equal to 11 nucleotides. According to this study estimates, *mF*_max_(11) = 592.0 and it corresponds to *P*(*mF*_max_ > 592.0) < 10^−50^, since the average value *mF*_max_ for random sequences* Sr* is ~127.8 and *σ* ~ 41.1. It means that the period equal to 12 nucleotides is the most statistically significant in this sequence. The resulting alignment and the resulting matrix* M*_0_ could be received from http://victoria.biengi.ac.ru/cgi-bin/indelper/index.cgi. A consensus periodicity equal to 12 nucleotides is CACAGTCTCAA(T/G).

We encountered multicity of sequences, where the tandem periodicity could only be detected using our developed method. Typically, these sequences could obtain the varying length periodicity, but *mF*_max_(*n*) < 600.0. Such sequences could be found at the http://victoria.biengi.ac.ru/indelper data bank. An average of 1.0 mutation per nucleotide is accumulated in these sequences and the periodicities contain a large number of insertions and deletions. In this case, statistically significant pairwise similarity between the periodicities could not be found. As a result, all developed methods are becoming “blind,” because they are based upon construction of the periodicities multiple alignments using the pairwise alignment thereof or the periodicity “germs” search, as is performed by the T-REKs program [21]. Our approach envisages the random position and weight matrices which appear to be the images of certain random multiple alignments, and we are changing those images to best fit the analyzed sequence (periods). That actually means that we are working immediately with the multiple alignment of tandem sequences (periods) in the form of position and weight matrices and are not using the pairwise alignments. Particularly this feature of our mathematical method allows finding the latent periodicity, that is, the tandem periods having a large number of base substitutions, insertions, and deletions present within each periodicity.

We also examined the periodicity identity, which was discovered by us in the* A. thaliana*,* C. elegans,* and* D. melanogaster* genomes. For these purposes, we obtained the frequency matrices for each sequence with the detected periodicity. To receive the frequency matrix we were using the created alignment. The frequency matrix demonstrates the number of each nucleotide* i* in each periodicity position* j*. The sum of the frequency matrix elements is equal to the length of the sequence minus the length of regions with insertions or deletions. In order to verify the matrices identity, we were using the distance between matrices, which was introduced as follows [43]:

(1) InMk,Ml=∑i=14nmki,jln⁡mki,j+∑i=14nmli,jln⁡mli,j−∑i=14nmki,j+mli,jln⁡mki,j+mli,j+skj+sljlnskj+slj−skjlnskj−sljlnslj.

Here *s*_*k*_(*j*) = ∑_*i*=1_^4*n*^*m*_*k*_(*i*, *j*), *s*_*l*_(*j*) = ∑_*i*=1_^4*n*^*m*_*l*_(*i*, *j*), and* n* is the periodicity length. 2*I*_*n*_ has an asymptotic Chi-square distribution with the degree of freedom df = 3(*n* − 1) [44]. *M*_*k*_ is the matrix from the first compared genome with the number* k* and *M*_*l*_ is the matrix from the second compared genome with the number* l*. Then using approximation of the normal distribution we have

(2) $$\begin{array}{c} X_{kl}=\sqrt{4I\left(M_{1},M_{2}\right)}-\sqrt{2df-1}. \end{array}$$

We are obtaining the value of *X*_*kl*_ ~ *N*(0,1). Then we calculated the minimum value of *X*_*k*_^min^ for each* k* from the first genome and for all* l* for from the second genome. *X*_*k*_^min^ = min*X*_*kl*_∀ *l*. The genomes are* A. thaliana*,* C. elegans,* and* D. melanogaster*, *k* ≠ *l*. As a result, we calculated the distribution *F*_*k*_(*x*) which is the number of matrices *N*_*k*_ from the first genome, which has *X*_*k*_^min^ > *x*. We also calculated *Fr*_*k*_(*x*) for pure random matrices. For this purpose, sequences with the length periodicity *n* from the first and second genome were randomly mixed and we determined therefore random matrices of 4 ×* n* size. The number of such matrices was fully consistent with the number of the found sequences and periodicities in the first and second compared genomes. Then we determined such a minimum value of *x* = *x*_0_, where *F*_*k*_(*x*_0_)/*Fr*_*k*_(*x*_0_) > 20.0. This ratio shows that the number of false positives is less than 5%. For each pair of various genomes from the multiplicity {*A. thaliana*,* C. elegans* and* D. melanogaster*} we determined *F*_*k*_(*x*_0_) and *Fr*_*k*_(*x*_0_). *F*_*k*_(*x*_0_) demonstrates the number of unique repeats characterizing only the first genome which could not be found in the second analyzed genome even as a single specimen. The *Fr*_*k*_(*x*_0_) values are shown in Table 1. The table row is the first genome for comparison and the table column presents the second genome to compare. This table demonstrates that* C. elegans* does not have any repeats, which are not discovered in the* A. thaliana* and* D. melanogaster* genomes. The* D. melanogaster* genome is characterized by greater difference relative to the* A. thaliana* genome. In this case we observed 391 tandem repeats, which were not found in the* A. thaliana* genome. On the other hand,* A. thaliana* obtains such types of tandem repeats that could not be found in the* elegans* and* D. melanogaster* genomes, although the number thereof is relatively small. These data generally show that tandem repeats that are specific for a particular genome could exist.

We studied the relationship between the detected tandem repeats and the known families of dispersed repeats. It was discovered in the* A. thaliana* genome that 3,740 regions out of the 10,191 regions found by us were associated with different dispersed repeats. In the* C. elegans* and* D. melanogaster* genomes these numbers are 2,578 out of 16,234 and 1,543 from 14,049, respectively. More detailed results covering the* D. melanogaster* and* C. elegans* genomes are presented in Table 2. These results testify that mostly sequences with periodicity could be found in the LTR (*D. melanogaster*) and retroposons DNA repetitive elements (*C. elegans*). The same results for human chromosomes (17–22) are presented in Table 3. It could be seen that such correlation is irregular in regard to the chromosomes, and the major part thereof is associated with LINE, SINE, or LTR.

It is also interesting to discuss the limits of applicability of the method developed in this study. As was noted above we used the local alignment of sequences* S*_1_ and* S*. The average value of the length for this alignment $\overset{-}{l}=200$ was chosen using the random sequences. This means that micro- and minisatellite sequences less than this length are detected being not sufficient for this method. The fact is that these lengths cannot overcome the threshold of* F*_0_ = 390.0; thus, these sequences could be missed when using this study method. This means that even perfect micro- and minisatellites may be skipped, if they obtain a length equal to or less than 200 nucleotides. On the basis of this limitation, a comparison could be made between the previous works on the micro- and minisatellite search and the results of this study. Previously, micro- and minisatellite sequences from* C. elegans* genome were investigated [45, 46] and mathematical methods for finding the micro- and minisatellites sequences were shown in [31]. The programs used included TRF [16], T-REKs [25], Mreps [22], BWTRs (Pokrzywa and Polanski, 2010), ATR hunter [24], and Repfind [41]. Therefore, it can be assumed that the developed approach misses micro- and minisatellite sequences, which obtain the length of less than 200 bases. However, the method used in this study was able to find a highly diverged periodicity sequence, which is characterized by considerable length (200 or more bases) and which passed the previously developed approaches. In this sense, this method was developed as an addition to the already developed techniques. Also, this study method is suitable when it comes to searching for highly divergent tandem repeats, characterized by a total length of more than 200 nucleotides.

We compared our results and methods with one of the recently published databases [47]. We selected out of the HeteroGenome database the sequences that obtain the length from 200 to 600 bases and the periodicity length from 2 to 50 and that are not characterized by the triplet periodicity, since under these conditions we conducted the tandem repeats search. The last condition is connected to the fact that many coding sequences obtain periodicity of the 3 bases length, which is not associated with the tandem repeats [37]. Therefore, these sequences were excluded from consideration. We studied the intersection of regions in our database (Indelper) and in HeteroGenome (Table 4). We constructed intersection with HeteroGenome under one setting: 30% overlap without periodicity length equality. The periodicity length (specified in HeteroGenome) was not considered due to the fact that the model sequences demonstrated inaccurate determination of the periodicity length in the HeteroGenome database [3]. Results of our comparison for the* A. thaliana*,* D. melanogaster,* and* C. elegans* genomes are shown in Table 1. The results demonstrate that more than 75% of sequences with latent periodicity submitted in the HeteroGenome database are also presented in the Indelper database. The rest of sequences are below the *F*_0_ = 390.0 threshold. This may be connected to the fact that HeteroGenome in some cases has sequences that contain regions with periodicities of varying lengths. An example of such a sequence could be found in the sequence of the 1st chromosome of* A. thaliana* 14264679–14264886, where artificially the length periodicities of 12, 13, 25, and 49 bases are combined [47]. This comparison also demonstrates that Indelper database contains significantly more (approximately 10 times) regions with periodicity than the HeteroGenome database. This is connected to the fact that the method used [47] does not allow detecting the tandem periodicity in the presence of symbols' deletions and insertions. In fact, the tandem periods become invisible for the method used in the HeteroGenome development, if even a single deletion or insertion is present therein. The performed comparison shows that the Indelper database contains a large number of tandem periodicities, which were not detected previously.

The results of this study were compared with those of the T-REKs program [25]. T-REKs program is one of the best tools for the tandem repeats search in DNA sequences. A comparison showed that the T-REKs program could detect not more than 30% sequences with periodicities found in this work at the false positives ratio ≤ 5%. There is a natural question about the biological significance of the periodicity found. Regions were found with periodicities that could be micro- and minisatellite sequences [45]. It is very likely that sequences with periodicity ranging from 9 to 11 bases are associated with the formation of chromatin loops and are found in this study [48]. There are earlier suggestions that the periodicity length of 10 and 11 nucleotides has a relationship with the *α*-helices in proteins, as well as with the processes of DNA compaction and protein binding [49, 50]. Early periodicity of 10 and 11 nucleotides was intensively studied in the study conducted by Trifonov et al. [49, 51–54].

**Figure 5:**
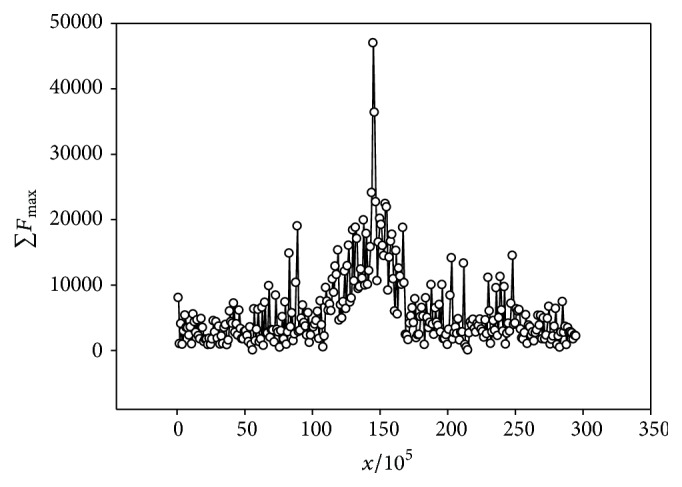
∑*F*_max_ for the chromosome 1 of the genome* A. thaliana*. *x* is the position in the chromosome. ∑*F*_max_ is the sum of *mF*_max_ for all sequences with periodicity which are found in the region with coordinates from (*x* − 1)/10^5^ to* x*/10^5^.

We studied the distribution of periodicity regions along the chromosome length of the* A. thaliana*,* D. melanogaster,* and* C. elegans* genomes. For this purpose we selected chromosomes segments of 10^5^ bases length and summarized in each segment the *mF*_max_ value. As a result, we got the ∑*F*_max_(*x*) value depending on *x*. The ∑*F*_max_ is an integral characteristic, which depends upon the number of periodicity regions in the segment of 10^5^ bases long as well as upon the level of evolutionary divergence of this periodicity. The higher the ∑*F*_max_ value is, the larger the number of periodicity regions is present and the higher the *mF*_max_ value is in the DNA segment 10^5^ bases long. The results of the analysis demonstrated availability of the two major ∑*F*_max_(*x*) dependence behavior models. The first model is typical for the chromosomes of the* A. thaliana* genome. ∑*F*_max_(*x*) typical dependence is shown in Figure 5. It could be seen that there is a clear maximum in the ~15 × 10^6^ nucleotide position. This maximum position correlates with the position of the centromere of the chromosome 1 from the* A. thaliana* genome [55]. The length of the region with the increased ∑*F*_max_ value constitutes about 5 × 10^6^ nucleotides, which is significantly more than the length of the found periodicity region with period equal to 180 nucleotides [55]. We studied periodicity within the interval from 2 to 50 nucleotides and the periodicity of 180 nucleotides long is detected in the form of periodicity that is the integer quotient from dividing 180 by whole numbers (<180) and that is less than 50 bases. A similar maximum correlation of the ∑*F*_max_(*x*) could be observed for the remaining chromosomes of the* A. thaliana* genome.

**Figure 6:**
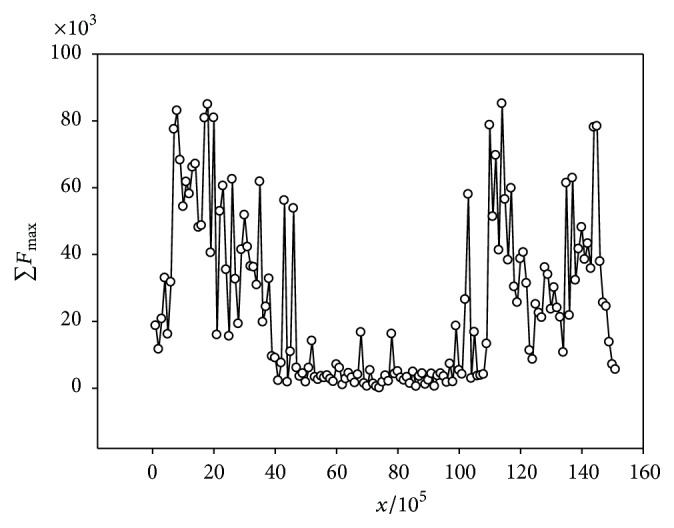
∑*F*_max_ for the chromosome 1 of the genome* C. elegans*. *x* is the position in the chromosome. ∑*F*_max_ is the sum of *mF*_max_ for all sequences with periodicity which are found in the region with coordinates from (*x* − 1)/10^5^ to* x*/10^5^.

The second model is shown in Figure 6 and is based on the first chromosome of the* C. elegans* genome example. In this case, within the centromere region, there is a slight increase of the ∑*F*_max_ values indicating the presence of a variety of the repetitive sequences. At the same time, a significant increase in the number of repetitive sequences could be observed close to the chromosome ends. The first model of the repetitive sequences distribution could be observed in the* D. melanogaster* genome and is shown in Figure 5. Only the increase in the repetitive sequences presence in the centromere region is expressed more weakly than in the* A. thaliana* genome. Probably these two models of the tandem repeats organization models are associated in some way with the two types of the centromere organization. Monocentric eukaryotes contain “localized” centromeres, where the centromere formation is restricted to a specific chromosomal locus. “Localized” centromeres are highly variable in size and sequence, including the simple “point” centromeres for the* S. pombe* budding yeast,* C. albicans* pathogenic fungus,* Drosophila*, plants, and human cells. On the other hand, in holocentric organisms, such as the* Caenorhabditis elegans* nematode, a “diffuse” centromere is forming along the entire chromosome [56]. It is very likely that the first model of the tandem repeats distribution along the chromosome associated with monocentric eukaryotes contains “localized” centromeres. The second model of the tandem repeats distribution is present in a diffuse centromere.

Our results are published at the http://victoria.biengi.ac.ru/indelper web database. It provides basic navigation in regard to the organisms and filtering by the length period and the periodicity significance score (*mF*_max_). Each periodicity sequence could be analyzed in detail by selecting it in the query results. The page will provide data on genomic position, statistical significance of the triplet periodicity (*Z*) [40], alignment against periodicities, periodicities consensus sequence, and periodicity weight matrix* M* [33]. The database we present here provides novel information on periodicity in DNA sequences by accounting for both indels and substitutions arisen during evolution. The current set of organisms in the database is a subject for further expansion by analyzing other genomes of interest.

## Figures and Tables

**Table 1:** Row of the table shows the number of unique tandem repeats, peculiar only to the specified genome. These repeats are not found in the second analyzed genome (column) even in the only instance.

	*A. thaliana*	*D. melanogaster*	*C. elegans*
*A. thaliana*	—	32	98
*D. melanogaster*	391	—	0
*C. elegans*	0	0	—

**Table 2:** The relationship at the location of known repeats and found periodicity regions in the genomes of *D. melanogaster* and *C. elegans*.

	*D. melanogaster*	*C. elegans*
Retroposon DNA repeat elements	60	1026
LINE	256	28
Low complexity	6	5
LTR	1079	69
rolling-circle (RC) transposons	81	562
rRNA	3	—
Satellite	253	484
Simple repeat	78	94
Other	12	—
Found in this work	14049	16234

**Table 3:** The relationship at the location of known repeats and found periodicity regions in the chromosomes 17–22 of the human genome.

	Chr.17	Chr.18	Chr.19	Chr.20	Chr.21	Chr.22
Retroposon DNA repeat elements	100	76	241	109	37	17
LINE	746	916	1817	1539	379	154
LTR	191	261	966	496	189	44
Rolling-circle (RC) transposons	2	—	—	—	2	—
Simple repeat	7	5	67	13	7	4
SINE	119	72	933	155	28	34
Low complexity	—	—	27	4	2	—
Found in this work	3127	3320	10680	5974	4073	2420

**Table 4:** Comparison of results of the Indelper and HeteroGenome databases. We compared periodicities with lengths from 2 to 50 bases and with region lengths from 200 to 600 bases. We excluded from consideration regions with triplet periodicity if *x* > 3.0 (see [37] for details).

	Indelper	HeteroGenome	Indelper∩HeteroGenome
*A*. *thaliana*	10190	633	465
*D. melanogaster*	16236	2190	2095
*C. elegans*	14049	620	547
